# Development of Two Mouse Models for Vaccine Evaluation against Cryptosporidiosis

**DOI:** 10.1128/iai.00127-22

**Published:** 2022-06-23

**Authors:** Denise Ann Dayao, Justyna Jaskiewcz, Sangun Lee, Bruno Cesar Oliveira, Abhineet Sheoran, Giovanni Widmer, Saul Tzipori

**Affiliations:** a Department of Infectious Disease and Global Health; Cummings School of Veterinary Medicine, Tufts University, North Grafton, Massachusetts, USA; b Center for Engineering in Medicine, Department of Surgery, Massachusetts General Hospital, Harvard Medical School, and Shriners Hospitals for Children, Boston, Massachusetts, USA; c União das Faculdades dos Grandes Lagos, São José do Rio Preto, Brazil; Albert Einstein College of Medicine

**Keywords:** Cryptosporidiosis, animal models, interferon gamma, *Cryptosporidium tyzzeri*, *Cryptosporidium parvum*, vaccine evaluation

## Abstract

Cryptosporidiosis was shown a decade ago to be a major contributor to morbidity and mortality of diarrheal disease in children in low-income countries. A serious obstacle to develop and evaluate immunogens and vaccines to control this disease is the lack of well-characterized immunocompetent rodent models. Here, we optimized and compared two mouse models for the evaluation of vaccines: the Cryptosporidium tyzzeri model, which is convenient for screening large numbers of potential mixtures of immunogens, and the Cryptosporidium parvum-infected mouse pretreated with interferon gamma-neutralizing monoclonal antibody.

## INTRODUCTION

Cryptosporidiosis has been reported in most mammalian species including humans, in which infection causes significant morbidity and mortality attributed to diarrheal disease in children younger than 2 years of age in low- to moderate-income countries (LMIC). The two parasite species most frequently associated with human disease are Cryptosporidium hominis and Cryptosporidium parvum, with the former often being responsible for a majority of cases in children in LMIC (1, 2). Cryptosporidiosis is the fifth leading cause of diarrhea in children younger than 5 years and second in children under the age of 2 (1). Cryptosporidiosis is also responsible for severe and life-threatening diarrhea and wasting in immunocompromised individuals with AIDS (3, 4) and transplant recipients (5). Currently, there are no effective drugs that can protect children and HIV/AIDS patients, nor are there vaccines available against the disease. The only FDA-approved drug, nitazoxanide, has limited efficacy in children and is not effective in immunocompromised individuals (6, 7).

Although the need for a vaccine is acknowledged by scientists and public health policy experts, serious challenges to develop and evaluate vaccines include the antigenic complexity of the parasite, the difficulty of *in vitro* cultivation and, more significantly, the lack of well-characterized and optimized immunocompetent rodent models in which to screen and identify putative protective antigen combinations and evaluate them for protective efficacy. Small rodents remain the most widely used models in research because of the availability of inbred strains, low cost, and availability of immunological reagents. Current *Cryptosporidium* mouse models used for drug discovery are either immunodeficient or immunosuppressed, neither of which are suitable for vaccine development, antigen selection, and evaluation. They include genetically modified mice, such as interferon gamma receptor knockout (IFN-γR-KO), interferon gamma knockout (IFN-γ-KO), interleukin 2 knockout (IL-2-KO), and severe combined immunodeficiency (SCID) mice (8–11). Immunocompetent rodent models previously reported include a murine model of infection with transgenic *C. tyzzeri* (strain UGA55) (12). Sateriale et al. (12) characterized elements of the protective immune responses and concluded that the model is suitable to evaluate therapeutics and vaccines. We also previously described (13) in some detail an immunocompetent rat model challenged intratracheally with C. hominis or C. parvum.

Here, we optimized and contrasted two murine models for screening parasite immunogens and mixtures thereof as potential vaccine candidates. The first model involves the use of the naturally occurring mouse pathogen *C. tyzzeri*, isolated in the Czech Republic (14). *C. tyzzeri* is distinct from the gastric murine species Cryptosporidium muris, which is confined to the gastric mucosa (15, 16). *C. tyzzeri* asymptomatically infects the small intestine of immunocompetent mice, causing villus blunting, crypt hyperplasia, and lymphocyte aggregation, as observed in most infected mammalian species with cryptosporidiosis (12). In the second model, immunized immunocompetent mice are treated with a single injection of IFN-γ-neutralizing monoclonal antibody (MAb) prior to challenge with C. parvum.

## RESULTS

### Optimization of the immunocompetent *C. tyzzeri* mouse model.

**FIG 1:**
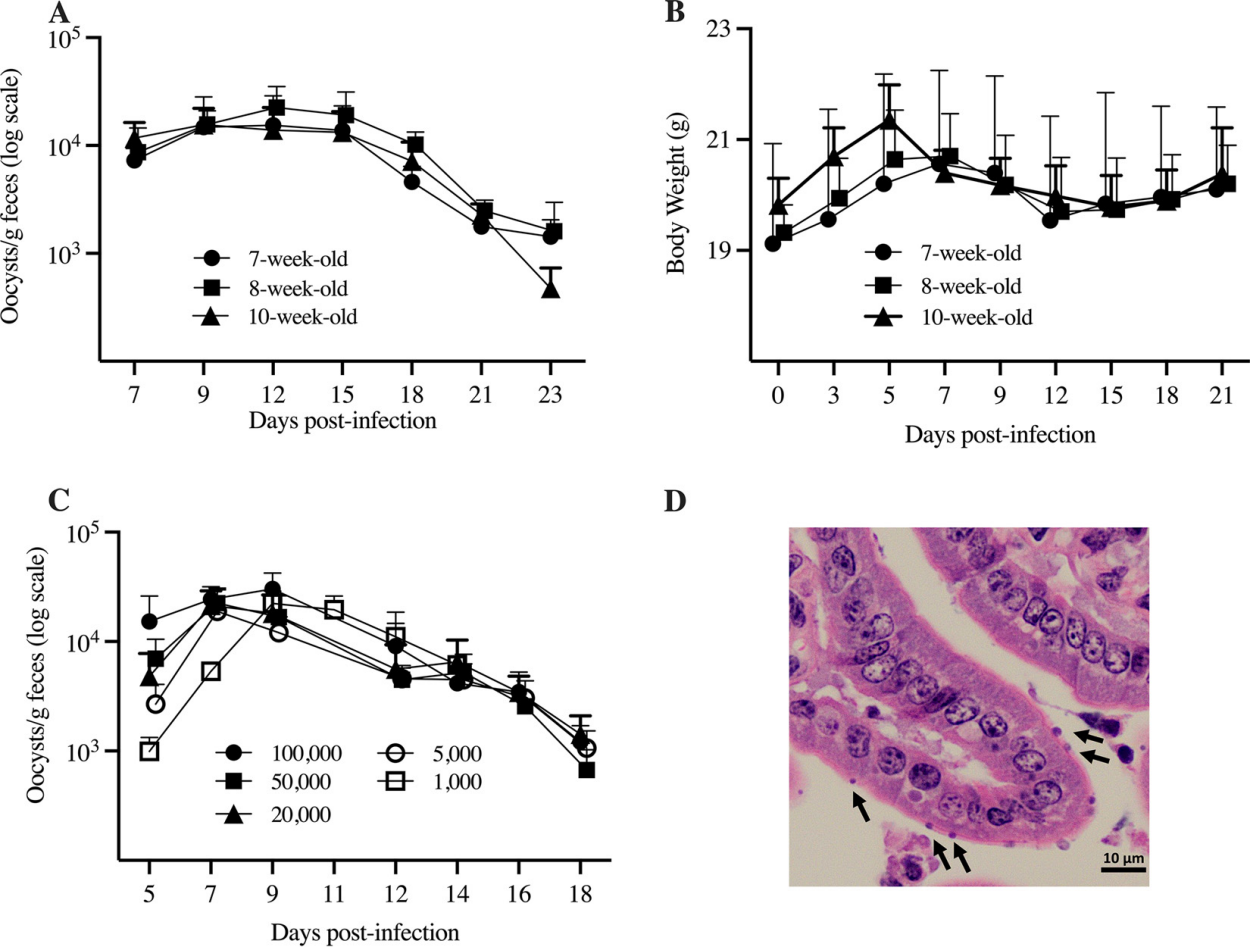
*C. tyzzeri* infection in C57BL/6 mice. (A) Pattern of *C. tyzzeri* oocyst shedding in 7-, 8-, and 10-week-old immunocompetent C57BL/6 mice (*n* = 5 animals per age group) orally infected with 100,000 oocysts. Data points represent oocyst concentrations (on a logarithmic scale) expressed as oocysts per gram of feces, as detected by flow cytometry. Mice were sampled individually. (B) Pattern of mean body weight fluctuation over the course of infection, expressed in grams (C) *C. tyzzeri* oocyst shedding by five mouse groups orally inoculated with decreasing doses of oocysts. Oocyst concentrations represent infection with 100,000, 50,000, 20,000, 5,000, or 1,000 oocysts. Groups 1 to 4 were sampled from 5 to 18 days postinfection (dpi) (*n* = 4). Group 5 data are from mean oocyst shedding from 5 to 14 dpi (*n* = 5). For all graphs, error bars show standard deviations. (D) *C. tyzzeri* in the small intestine 7 days after infection of immunocompetent mice. The micrographs are of hematoxylin and eosin-stained jejunal sections from C57BL/6 mice. Arrows indicate intracellular parasite stages in the apical region of largely intact enterocytes. Scale bar, 10 μm.

**(i) Age susceptibility.** C57BL/6 mice infected with *C. tyzzeri* at ages 7, 8, and 10 weeks excreted oocysts in feces between 5 and 23 days postinfection (dpi), as measured at seven time points (Fig. 1A). A total of 105 (35 per group) fecal samples collected over 7 time points over 19 days were analyzed for parasite load by flow cytometry (Fig. 1A). For all groups, oocyst shedding peaked between 7 and 15 dpi and decreased in the third week of infection. Fecal smears from individual mice collected on day 23 postinfection were all negative by immunofluorescent microscopy. Cumulatively, over all days, mean oocyst counts of 59,125, 80,197, and 64,390 oocysts/g feces were measured for 7-, 8-, and 10-week-old mice, respectively. There were no statistically significant differences in fecal oocyst burden between the three age groups (Kruskal-Wallis test, *P = *0.6386). All mice were apparently healthy, with body weight only transiently decreasing during peak shedding, suggesting a clinical effect of the infection. Weight was restored by the third week of infection (Fig. 1B).

### (ii) Dose response.

All mice infected with different doses of *C. tyzzeri* excreted oocysts from 5 to 18 dpi. Mice infected with 1,000 shed oocysts starting on 5 dpi until euthanasia. Oocyst shedding by all groups showed only minor differences in levels of parasite excretion (Fig. 1C). Figure 1D shows the robust infection with *C. tyzzeri* in the small intestines of immunocompetent C57BL/6 mice at 7 dpi. 

### (iii) Mouse strain susceptibility.

All mice primed with 10^5^ *C. tyzzeri* oocysts were protected when challenged with 10^3^ *C. tyzzeri* oocysts 4 weeks later. No mice shed oocysts in feces from 4 to 19 dpi as determined by immunofluorescence microscopy and flow cytometry. We found no differences in the response to infection between the BALB/c and C57BL/6 mouse strains tested.

### Protection in mice immunized with C. hominis mRNA to challenge with *C. tyzzeri*.

**FIG 2:**
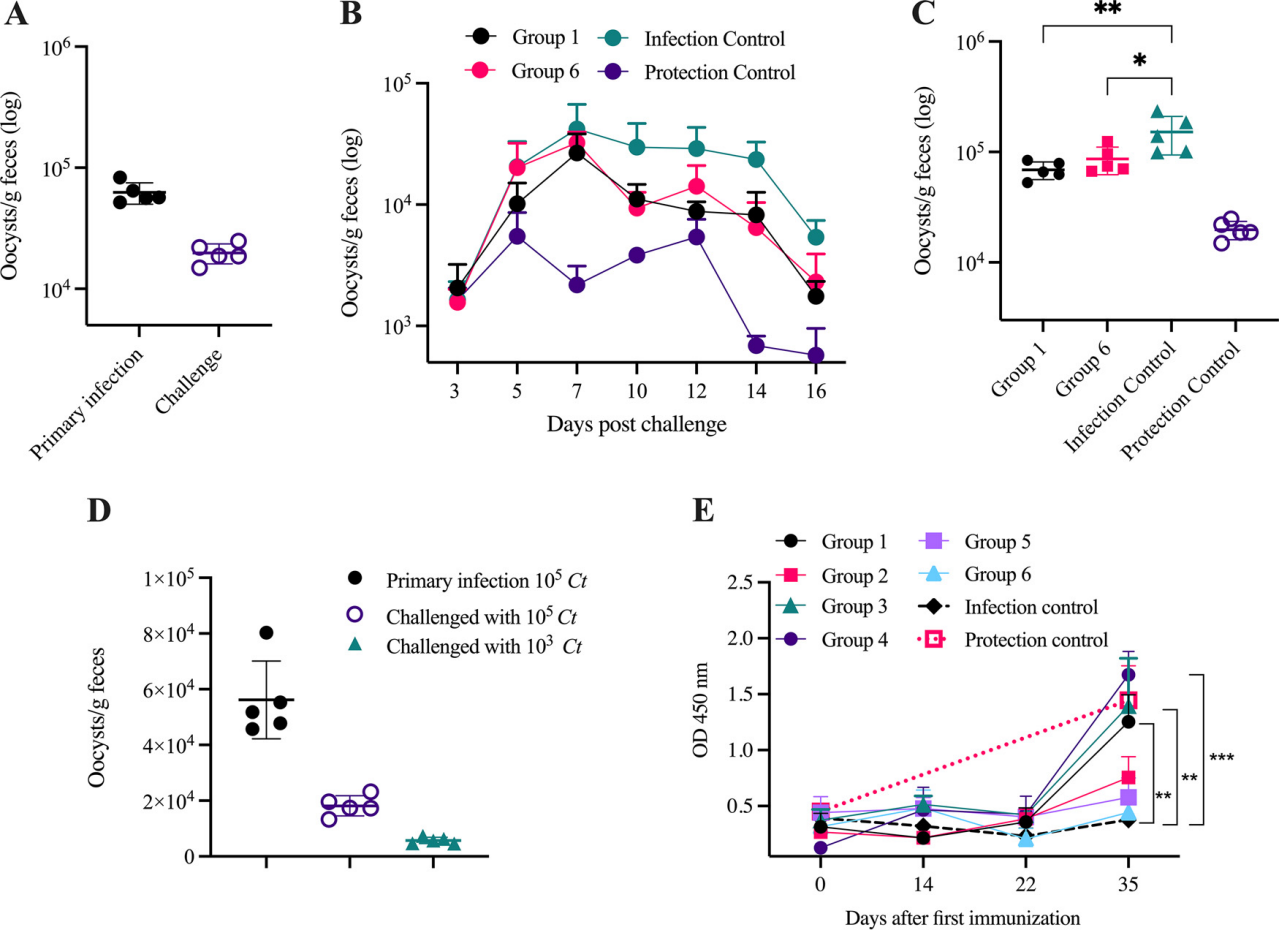
Effect of mRNA immunization on oocyst excretion of *C. tyzzeri* and fecal *Cryptosporidium-*specific IgA in mice. C57BL/6 mice were immunized 3 times at 2- to 3-week intervals with mRNAs and *C. tyzzeri* oocysts as indicated in Table 1. Two weeks after the third immunization, mice were challenged orally with 10^5^ *C. tyzzeri* oocysts. For log oocyst concentrations, data are expressed as number of oocysts per gram of feces, quantified by flow cytometry. For all groups, 5 mice were individually sampled on 7 time points 3–16 days post primary infection and postchallenge. (A) Protection control mice: average parasite output by each mouse after primary and challenge infections. (B) Mean oocyst shedding by groups 1 and 6, the infection and protection control mice immunized with mRNA combinations as indicated in Table 1. (C) Comparison of oocyst output of immunized and control mice. mRNA-immunized mice shed fewer oocysts than the infection control (Dunnett’s multiple-comparison test; group 1, *P = *0.0024; group 2, *P = *0.0141). Values are cumulative counts of oocysts shed by each mouse on 7 collection time points between 3 and 16 dpi. (D) The cumulative *C. tyzzeri* oocyst output (6 time points, at 5 to 16 dpi), indicating that the 5 C57BL/6 mice that were orally primed with 10^5^ *C. tyzzeri* oocysts were protected when challenged with 10^3^ *C. tyzzeri* oocysts 4 weeks later. (E) Fecal *Cryptosporidium-*specific IgA in mice. Samples were collected on days 0 (before the first immunization), 14 (before second immunization), 22 (before third immunization), and 35 before oral challenge with *C. tyzzeri* oocysts. Values indicate optical densities (OD) measured at 450-nm absorbance. On day 35, protection controls and those immunized with mRNA combinations 1, 3, or 4 had significantly higher *Cryptosporidium*-specific IgA than mice immunized with irrelevant mRNA (one-way ANOVA with Dunn’s multiple-comparison test; *P = *0.0061, 0.005, and <0.0001, respectively). For all graphs, values represent the means for five mice. Error bars show SD.

An experiment was performed to determine whether C. hominis-derived mRNAs protect C57BL6 mice from *Cryptosporidium* challenge (Table 1). Six groups of 5 mice (groups 1 to 6) were immunized intradermally (i.d.) with different mRNA combinations. Five mice were immunized with irrelevant mRNA (group 7, infection control), and 5 mice were infected with a primary dose of 10^5^ *C. tyzzeri* oocysts (group 8, protection control) . Beginning 3 days post-primary infection, feces from group 8 mice were analyzed for the presence of oocysts. All five mice in this group shed oocysts from 4 to 16 days post-primary infection. Two weeks after the last immunization, mice were challenged orally with 10^5^ *C. tyzzeri* oocysts. Mice from all 8 groups excreted oocysts after challenge, as determined by immunofluorescent microscopy and quantified by flow cytometry. The *C. tyzzeri*-infected control mice (group 8) were not fully protected after the second challenge, as they continued to shed oocysts, although significantly fewer than during the primary infection (Mann-Whitney test, *P = *0.0023) (Fig. 2A). The 6 mixtures of three to four mRNA combinations generated from 15 mRNA transcripts encoding C. hominis antigens were selected based on reverse vaccinology, immunoproteomics, and genetic homology with other enteric apicomplexan antigens (Table 1). Only 2 of the 6 mRNA mixtures (administered to groups 1 and 6) reduced oocyst shedding when animals were challenged 2 weeks after the third i.d. mRNA immunization, compared with that in control group 7 (Dunnett’s multiple-comparison test, group 1: *P = *0.0024; group 6: *P = *0.0141) and group 8, as shown in Fig. 2B and C. The pattern of oocyst excretion of the other four mixtures of mRNAs (groups 2 to 5) were indistinguishable from that in the control mice of group 7 immunized with irrelevant mRNA (see Fig. S1A in the supplemental material). Our findings show that mRNA administration provided some protection against challenge with *C. tyzzeri* in groups 1 and 6.

**TABLE 1:** Components of mRNA vaccines^*a*^

Mouse group	mRNA vaccine components^*b*^
1	Chro. 80295 Sushi domain, Chro. 60138 sporozoite antigen gp40/15, Chro. 20173 fibrillin-2, Chro. 50022 hypothetical protein
2	Chro. 20320 hypothetical protein, Chro. 50232 hypothetical protein, Chro. 70565 NIMA-related kinase 5, cgd7_4020 C-terminal fragment of mucin-like glycoprotein gp900
3	cgd8_4830 hypothetical protein, Chro. 70165 SbmA/BacA-like protein, cgd7_550 hypothetical protein, Chro. 40312 hypothetical protein
4	Chro. 10296 calcium antiporter, Chro. 60544 multipass transmembrane protein, Chro. 80295 Sushi domain, Chro. 60138 sporozoite antigen gp40_15
5	Chro. 20173 fibrillin-2, Chro. 50022 hypothetical protein, Chro. 20320 hypothetical protein, Chro. 50232 hypothetical protein
6	Chro. 40414 immunodominant antigen Cp23, Chro. 60138 sporozoite antigen gp40_15, cgd7_4020 C-terminal fragment of mucin-like glycoprotein gp900
7	Orally primed with 10^5^ *C. tyzzeri* oocysts at onset of expt
8	Irrelevant mRNA encoding Photinus pyralis luciferase

a Six groups of C57BL/6 mice immunized i.d. three times with mixtures of selected mRNA transcripts encoding C. hominis genes were compared with two control mouse groups, groups 7 and 8 (included as the positive and negative controls, respectively). All 8 groups were challenged after the third i.d. immunization with 10^5^ *C. tyzzeri* oocysts.

b Gene IDs are according to cryptoDB.org; IDs that include “Chro” originated from C. hominis TU502 (40); IDs designated with cdg originated from Cryptosporidium parvum IOWA (41).

We found that protection control mice (group 8) primed with 10^5^ *C. tyzzeri* oocysts were not fully protected against a challenge with the same dose of *C. tyzzeri* oocysts. This observation led us to try a lower secondary challenge dose of 10^3^, against which the primed mice were completely protected (Fig. 2D), offering a more sensitive option to evaluate immunogens.

To determine whether mRNA immunization induced humoral responses, we tested feces and sera from each mouse for *Cryptosporidium*-specific antibodies. IgA was measured in feces collected before each immunization on days 0, 14, and 22 and before challenge on day 35. Only groups 1, 3, and 4 had elevated fecal IgA prior to oral challenge (day 35) compared to group 7 (Dunn’s multiple-comparison test, *P = *0.0061, 0.005, and <0.0001, respectively) (Fig. 2E). No serum IgG was detectable by enzyme-linked immunosorbent assay (ELISA) for any of the mRNA-immunized or control animals (see Fig. S1B).

### Establishing a C. parvum mouse model for evaluation of immunogens.

**FIG 3:**
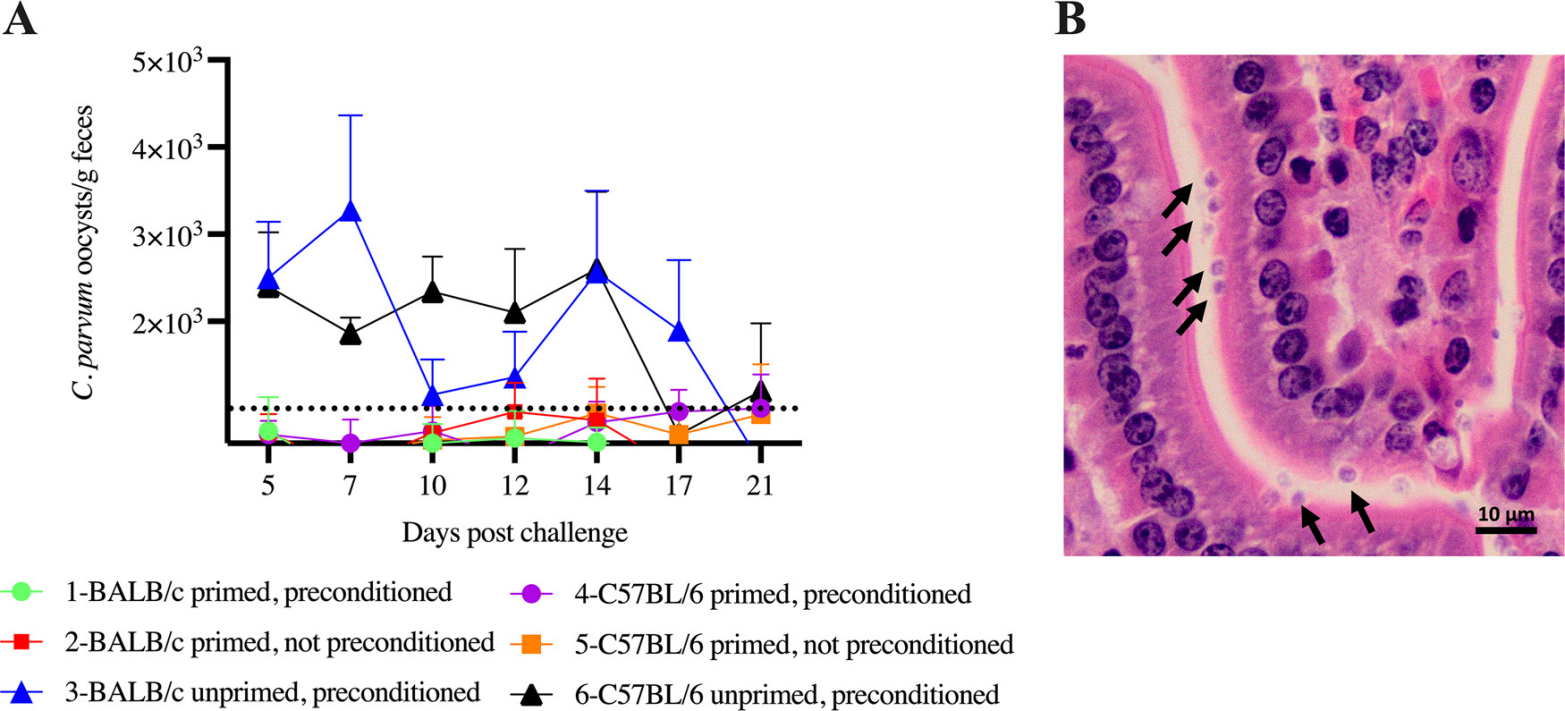
Effects of primary infection and IFN-γ suppression on oocyst excretion by C. parvum-challenged mice. The graphs show the concentration expressed as the number of oocysts per gram of feces, quantified by flow cytometry (FCM). Mice were sampled individually on seven time points between 5 and 21 days postchallenge (*n* = 5 animas per group). (A) Mice that received a primary C. parvum infection showed no apparent infection regardless the status of preconditioning with IFN-γ-neutralizing MAb (groups 1, 2, 4, and 5). In contrast, the unprimed mice preconditioned with IFN-γ-neutralizing MAb became heavily infected after the secondary challenge (groups 3 and 6). Feces from uninfected mice served as negative control samples. The dashed line represents FCM background counts obtained with these controls. Error bars show SD. (B) C. parvum in the small intestine 7 days after infection of immunocompetent mice. Micrographs are of hematoxylin and eosin-stained jejunal sections from a C57BL/6 mouse treated with IFN-γ-neutralizing MAb and infected 2 h later with C. parvum. Arrows indicate intracellular parasite stages in the apical region of largely intact enterocytes. Scale bar, 10 μm.

Table 2 summarizes the design of an experiment that showed that BALB/c and C57BL/6 mouse strains were equally and completely resistant to infection with 10^6^ oocysts of C. parvum (groups 1, 2, 4, and 5). However, Table 2 and Fig. 3 show that intraperitoeal (i.p.) injection of IFN-γ-neutralizing MAb 2 h prior to oocyst challenge rendered the mice temporarily susceptible to C. parvum (groups 3 and 6). Figure 3B shows intracellular stages of C. parvum in the small intestine of an immunocompetent mouse on day 7 postinfection. Table 2 and Fig. 3A further show that, unlike the *C. tyzzeri* mouse model, priming mice with C. parvum, despite the absence of apparent infection, protected them against a high dose of a second challenge 4 weeks later. This immunoprotection in the C. parvum model against secondary challenge compares favorably with the *C. tyzzeri* model for assessing the immunogenic efficacy of parasite antigens necessary for vaccine development.

**TABLE 2:** Evaluation of the impact of parenteral treatment with IFN-γ-neutralizing MAb in 3 groups of BALB/c and 3 groups of C57BL/6 prior to challenge with C. parvum

Group^*a*^	Mouse strain	Prime/challenge C. parvum doses	IFN-γ-neutralizing MAb (1 mg/mouse)	Oocyst shedding^*b*^ after challenge
1	BALB/c	10^6^*/*10^6^	+	−
2	BALB/c	10^6^*/*10^6^	−	−
3	BALB/c	*—/*10^6^	+	+
4	C57BL/6	10^6^*/*10^6^	+	−
5	C57BL/6	10^6^*/*10^6^	−	−
6	C57BL/6	*—/*10^6^	+	+

a Groups 1, 2, 4, and 5 were primed with 10^6^ C. parvum. All 6 groups were challenged 4 weeks after the primary dose with 10^6^ C. parvum oocysts.

b Fecal oocyst shedding was monitored by immunofluorecent microscopy and quantified by flow cytometry.

### Outcome of systemic immunizations of mice with C. hominis and C. parvum oocyst lysates against C. parvum challenge.

**FIG 4:**
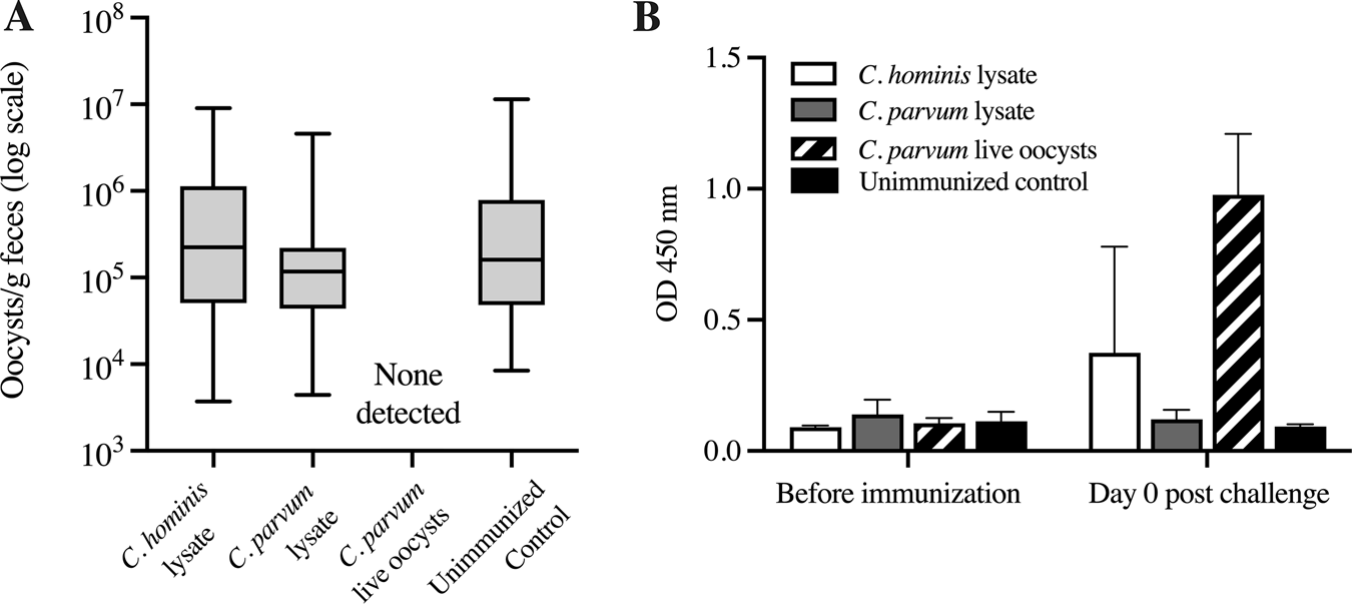
The outcome of three intraperitoneal immunizations with C. hominis or C. parvum lysates in mice receiving IFN-γ-neutralizing MAb prior to challenge with C. parvum. Oocyst excretion (A) and levels of *Cryptosporidium*-specific IgA (B) were measured in the feces by qPCR and ELISA, respectively. BALB/c mice were challenged with C. parvum after immunization with lysed C. hominis or C. parvum and compared with a control group orally immunized with live C. parvum and another control group of unimmunized mice. (A) Distribution of oocyst output measured at nine time points between 3 and 15 days postchallenge, measured by qPCR. Data indicate numbers of parasite copies detected per gram of feces. (B) Level of *Cryptosporidium*-specific IgA detected in feces before and after immunizations (day 0 postchallenge). Values indicate the OD measured at 450-nm absorbance. Mice were sampled individually; *n* = 5 per immunized group and *n* = 4 for the unimmunized group. Error bars show SD.

The goal of this experiment was to determine whether systemic administrations of lysed oocysts of C. hominis or C. parvum protect mice against a subsequent challenge with C. parvum. Mice received three systemic immunizations with oocysts that had been bleached, washed, and lysed by repeated cycles of freeze-thawing. Thereafter, mice were treated i.p. with 1 mg of IFN-γ-neutralizing MAb 2 h prior to oral challenge with 10^6^ C. parvum oocysts. The parasite load in feces was determined by quantitative PCR (qPCR) (Fig. 4A). All mice except for the orally primed control (group 3) were positive by PCR targeting the COWP gene, beginning at 3 to 4 days postchallenge. No significant differences were found between oocyst output in the groups immunized with lysates and that from the orally challenged control mice (group 4) (one-way analysis of variance [ANOVA] with Dunn’s multiple-comparison test), indicating that systemic immunization with lysed oocysts does not protect against C. parvum challenge when mice are pretreated with IFN-γ-neutralizing MAb. The orally primed control mice, in contrast, were negative by qPCR, COWP PCR, and acid-fast staining, which indicated that primary infection delivered robust protection against the secondary challenge even when IFN-γ activity was temporarily neutralized. This observation is consistent with the result we obtained in experiment 3, in which mice orally primed with C. parvum oocysts were protected against a subsequent challenge.

*Cryptosporidium*-specific IgA and IgG levels in feces and serum, respectively, were determined by ELISA. Serum IgG increased in mice immunized with lysate of C. parvum oocysts (group 2) after the second immunization, compared with other groups (see Fig. S2A in the supplemental material). This suggests that elevated systemic IgG did not protect against C. parvum challenge. In contrast, at the time of the challenge, IgA levels were higher in feces of the orally primed control group (group 3) than in those of unimmunized control group (Mann-Whitney test, *P* = 0.0159) (Fig. 4B). Group 3 mice did not shed oocysts after challenge and were negative for COWP PCR; these findings suggest that the level of specific IgA produced after the primary infection with live C. parvum oocysts provided significant protection from secondary challenge. Mice immunized systemically with oocyst lysates (groups 1 and 2) produced low levels of specific IgA during the immunization phase (see Fig. S2B) and before the challenge (Fig. 4B).

## DISCUSSION

Our focus over the last few years has been the development of a vaccine against cryptosporidiosis, one of four major enteric pathogens that contribute to morbidity and mortality of children under 2 years of age in LMIC (1). While vaccines for 3 of these 4 enteric pathogens are available (for rotavirus) or are in clinical trials (for E. coli and *Shigella*), vaccination against cryptosporidiosis is still an elusive goal. A major obstacle, among many, to vaccine development against this pathogen has been the lack of sensitive, well-characterized, and optimized immunocompetent small animal models in which to screen and characterize potential individual and combination immunogens necessary for the evaluation of vaccine efficacy.

We previously described an immunocompetent intratracheal rat model which has valuable properties, such as its susceptibility to C. hominis and C. parvum, but has a narrow scope, limiting its large-scale use (13). In this communication, we describe the optimization of two immunocompetent mouse models.

Due to their immunological features, we have selected BALB/c and C57BL/6 to determine which mouse strain displays greater responsiveness to a primary parasite challenge. BALB/c mice are characterized by a predominant humoral immune response mediated by type 2 T helper cells under physiological conditions or during stress or antigenic stimulation. By contrast, C57BL/6 mice are genetically predisposed to the predominance of Th1-mediated cellular immunity (17, 18). We observed no notable difference between the two mouse strains in their response to *Cryptosporidium* infections, with or without treatment with IFN-γ-neutralizing MAb. Reducing the secondary challenge dose from 100,000 to 1,000 oocysts considerably improved the sensitivity (as seen in Fig. 2D) of the *C. tyzzeri* mouse as a model for screening potential individual and mixtures of immunogens.

The *C. tyzzeri* mouse model, regardless of mouse strain, age, and parasite dose, proved to be highly susceptible, displaying no apparent symptoms, despite the high level of infection, apart from a slight and brief drop in body weight. *C. tyzzeri*-infected mice excreted oocysts for at least 21 days after the primary challenge.

The focus of this communication is the characterization and optimization of rodent small models and less about vaccine development and testing *per se*. To demonstrate the utility of the *C. tyzzeri* model, we evaluated mixtures of mRNAs given i.d. for their ability to protect against parasite challenge. The 15 mRNAs in Table 1 were selected based on reverse vaccinology (19), immunoproteomics, homology to the Eimeria tenella genes (ETH_00007985 and ETH_00039940), and evidence of antigenicity (gp40_15, gp900, and Cp23). Selecting homologs of the *Eimeria* genes was intended to take advantage of a better-studied apicomplexan pathogen and advances in the development of vaccines against coccidosis. Groups 1 and 6, immunized with mixtures of three to four C. hominis mRNAs, showed evidence of reduced oocyst excretion compared with mice immunized with an irrelevant mRNA. This effect was also reflected in fecal IgA production in mice of group 1 compared with controls. However, we found undetectable serum IgG. The finding of induced fecal IgA but not systemic IgG is inconsistent with other studies in which mRNA and i.d. immunizations induce both systemic and mucosal (i.e., IgA in feces, saliva) immune responses (20–22). As for the other mRNA-immunized groups, it was not possible to determine whether the choice and formulation of mRNAs provided little or no protection at all or whether the lack of protection was due to limited model sensitivity when challenges contained high *C. tyzzeri* oocyst doses. We will continue to test other mRNA combinations, as mRNAs have emerged as a highly effective platform to deliver vaccine antigens and therapeutic proteins (20). The challenge of delivering vaccines or therapeutics directly into the gastrointestinal tract to stimulate a local mucosal response, however, remains a serious challenge.

The resistance of immunocompetent mice to infection with C. parvum has been documented extensively in the literature and led us to first explore the use of an IFN-γ-deficient model for drug evaluation (8). The next step was to use IFN-γ-neutralizing monoclonal antibody to render immunocompetent mice temporarily susceptible to C. parvum (23, 24). In this study, we leveraged these observations to develop the current C. parvum mouse model treated with IFN-γ-neutralizing MAb prior to challenge, with the goal to screen potential immunogens for the evaluation of mRNA mixtures in the C. parvum model. IFN-γ-neutralizing MAb temporarily blocks IFN-γ, has an *in vivo* half-life of 3 to 4 days, and will be cleared within 2 to 3 weeks (24). We acknowledge that IFN-γ is required to prevent initiation as well as to limit the extent of *Cryptosporidium* infection, and blocking its activity could briefly mask an IFN-γ-associated protective effect of vaccine candidates. However, protection from *Cryptosporidium* challenge involves other protective immune responses (cellular and humoral) (25–28). In addition, protection can be achieved in the absence of IFN-γ, as reported for IFN-γ-KO mice, which mounted prolonged IgA and IgG responses after primary infection and did not shed oocysts after challenge (29). The C. parvum model proved to be considerably more sensitive, with animals resisting a secondary challenge; we consider this to be a significant attribute for testing the immunogenic nature of antigens.

With these models, we also aim to explore the mucosal route of vaccine delivery. Mucosal immunizations are known to elicit more effective immune responses against enteric infections than systemic immunization routes (30). High levels of mucosal *Cryptosporidium* Cp23-specific IgA were reported to delay parasite reinfection in young children and to protect them from diarrhea-associated growth faltering. This partial immunity is believed to be associated with acquired mucosal IgA (31, 32). Similarly, oral immunization with live C. parvum oocysts stimulated *Cryptosporidium-*specific IgA in feces and mice became more resistant to a secondary challenge even after treatment with IFN-γ-neutralizing MAb. This observation is in contrast with the results obtained with mice systemically immunized with lysed oocysts. Many studies in animals and humans, however, have demonstrated the roles of cell-mediated responses (CD4^+^ and CD8^+^ cells) and humoral immune responses, such as IgA, IgG, IgM, and protection against cryptosporidiosis conferred by hyperimmune colostrum (25–28). Although not measured in these experiments because of the experimental design, the degree of mucosal lesions and parasite distribution in the gut could potentially provide additional parameters to measure the extent of protection, or lack thereof, after immunization.

In conclusion, we describe here two mouse models in which to screen and identify immunogens against cryptosporidiosis with a view to construct an effective vaccine. Although *C. tyzzeri* is genetically and antigenically close to C. parvum and C. hominis, the species differ substantially in their host predilection. Our plan is therefore to use the *C. tyzzeri* model for screening potential immunogens and evaluate mixtures thereof as potential vaccine candidates in the C. parvum mouse model. Unlike *C. tyzzeri*, C. parvum has a broad mammalian host spectrum, including humans, and is genetically closely related to C. hominis, which vaccines should target.

## MATERIALS AND METHODS

### Parasites.

A naturally occurring *C. tyzzeri* strain isolated from a house mouse (Mus musculus) in the Czech Republic (14) was kindly provided by Martin Kváč (Biology Centre CAS, Czech Republic). The isolate was serially passaged in C57BL/6 mice at Tufts University. Oocysts were purified from feces on Nycodenz step gradients (Alere Technologies AS, Norway) as previously described (33) and stored at 4°C. The zoonotic C. parvum IOWA strain was obtained from Bunchgrass Farm (Deary, ID, USA). Before inoculation, oocysts were treated with 10% bleach (0.5% sodium hypochlorite) for 7 min on ice to eliminate bacterial contaminants, followed by three washes with distilled water and centrifugation at 18,000 × *g*. In experiment 4, lysed oocysts were used to immunize mice. Oocysts were bleached and washed as described above and then lysed by seven cycles of freezing in liquid nitrogen and thawing at 37°C.

### Animals.

Female C57BL/6 or BALB/c mice (Charles River Laboratories, USA) were housed in bedded cages in randomly assigned groups of four to five per cage with access to food and water *ad libitum*. Mice were infected orally using a pipette tip with freshly bleached and washed *Cryptosporidium* oocysts in 20 μL sterile distilled water. Blood samples were collected from the facial vein. Mice were euthanized by carbon dioxide asphyxiation, followed by cervical dislocation.

### Mouse experiments.

**Optimizing the immunocompetent *C. tyzzeri* mouse model. (i) Age susceptibility.** To determine the age susceptibility, 7-, 8-, and 10-week-old immunocompetent C57BL/6 mice (5 per age group) were challenged orally with 100,000 *C. tyzzeri* oocysts. Oocyst shedding in feces was monitored by fluorescence microscopy and measured by flow cytometry.

### (ii) Infectious dose.

To determine the optimal infectious dose of *C. tyzzeri*, 5 groups of C57BL/6 mice, five mice per group, age 11 weeks old, were infected with 100,000, 50,000, 20,000, 5,000, or 1,000 *C. tyzzeri* oocysts, respectively.

### (iii) Mouse strain susceptibility.

To determine strain susceptibility, 8- to 9-week-old BALB/c mice were compared with age-matched C57BL/6 mice. Five mice in each group were orally primed with 10^5^ *C. tyzzeri* oocysts followed by a secondary infection with 10^3^ oocysts 4 weeks later, and the pattern and intensity of oocyst excretion were monitored after each challenge.

### Protection in mice immunized with mixtures of C. hominis-derived mRNAs challenged subsequently with *C. tyzzeri*.

Six groups of five C57BL/6 mice, age 3 to 5 weeks, were immunized i.d. with different mRNA preparations (groups 1 to 6) (Table 1). The mRNAs were generated by CureVac (Germany). Control mice were immunized either with an irrelevant mRNA (group 7) or infected with 10^5^ live *C. tyzzeri* oocysts at onset of immunization (group 8). The i.d. immunization was achieved using syringe with a 30-gauge needle. Fecal shedding of oocysts in the infected positive control animals was monitored, and oocysts were enumerated from day 5 postinfection onwards using flow cytometry. The mRNA combinations in sterile saline solution (20 μg of each mRNA) were injected three times, at 2- to 3-week intervals. Mice were injected i.d. in two sites (50 μL per site) on the back while under anesthesia with a ketamine and xylazine cocktail (50 to 100 mg/kg and 5 to 10 mg/kg, respectively), given intraperitoneally (i.p.). At 2 weeks after the last mRNA immunization, all mice were orally challenged with 10^5^ *C. tyzzeri* oocysts, including the controls. Oocyst shedding in feces was monitored and measured by immunofluorescent microscopy and flow cytometry.

### Establishing the mouse immunization model of C. parvum pretreated with anti-IFN-γ MAb prior to parasite challenge.

Three groups of BALB/c mice (groups 1 to 3) and 3 groups of C57BL/6 mice (groups 4 to 6), with 5 mice per group, age 3 to 4 weeks, were treated as summarized in Table 2. Pooled fresh feces from each experimental group were examined daily for oocyst shedding by immunofluorescent microscopy, starting 3 days post-primary infection. Four weeks after priming, groups 1, 3, 4, and 6 were preconditioned with a single i.p. injection of an IFN-γ-neutralizing rat anti-mouse MAb at 1 mg/mouse (24). Two hours later, all mice were challenged orally with 10^6^ C. parvum oocysts. Shedding of oocysts in feces was monitored by immunofluorescent microscopy and analyzed by flow cytometry from 5 to 21 days postchallenge.

### Intraperitoneal immunization of mice with C. parvum or with C. hominis oocyst lysates.

Four groups of 3- to 4-week-old BALB/c mice, 4 to 5 mice mice per group, were assigned as follows. Group 1 was administered i.p. with lysate of 10^6^ C. hominis oocysts; group 2 was administered i.p. with lysate of 10^6^ C. parvum oocysts; controls (group 3) were orally infected with 10^6^ C. parvum oocysts; group 4 was infected at the time of challenge of groups 1 to 3. Mice in groups 1 and 2 were immunized i.p. three times with oocyst lysates at 2-to 3-week intervals. Blood and feces were collected for antibody detection from each mouse prior to each procedure. The i.p. injections of IFN-γ-neutralizing MAb were given to the 4 groups 2 h before oral challenge with 10^6^ C. parvum oocysts. Oocyst shedding of individual mice was monitored by modified acid-fast staining, PCR, and qPCR.

### Monitoring of oocyst shedding.

Starting 3 to 5 days postchallenge, fecal shedding of oocysts was monitored once per day by microscopy (modified acid-fast staining or immunofluorescence) or COWP PCR (experiment 4) and quantified by flow cytometry (experiments 1 to 3) or qPCR (experiment 4). To collect feces for oocyst quantification by flow cytometry, mice were placed individually into collection cages fitted with a wire bottom for overnight collection. Feces were stored at 4°C. Oocysts were enumerated as previously described (34) using in-house-produced MAb 5F10 specific to the *Cryptosporidium* oocyst wall (35, 36). Briefly, processed fecal samples from individual mice were incubated with 5F10 MAb cell culture supernatant at a 1:5 dilution in phosphate-buffered saline (PBS) and labeled with Alexa Fluor 488-conjugated goat anti-mouse IgG (catalog number A11029, Invitrogen, USA) at 1:500 dilution. Labeled samples were analyzed using a Becton Dickinson Accuri C6 cytometer.

For microscopy, samples were prepared from one or two pellets of fresh feces that were homogenized in sterile distilled water. Thin fecal smears were prepared on microscope slides, air dried, heat fixed, and processed for modified acid-fast staining (37) or for immunofluorescent microscopy. For immunofluorescent detection, the smears were first blocked with 10% fetal bovine serum, then incubated for 15 min with MAb 5F10 (cell culture supernatant at 1:5 dilution in PBS), followed by 15-min incubation with Alexa Fluor 488-conjugated goat anti-mouse IgG (catalog number A11029, Invitrogen, USA) diluted 1:500 in PBS, including three PBS washes between each step. Slides were washed with PBS a final time, air dried, and observed under fluorescence microscope at 400× magnification for the presence of oocysts.

In experiment 4, in addition to microscopy, COWP PCR was used to monitor oocyst shedding. qPCR was performed to measure fecal parasite load. Genomic DNA was extracted from freshly collected feces from individual mice by using the QiaAmp Fast DNA stool minikit (Qiagen, USA, catalog number 51604). COWP PCR was performed as previously described (38). qPCR was based on detection of copies of the *Cryptosporidium* heat shock protein gene (HSP70) (39) and completed using a Step-One Plus instrument (Applied Biosystems, USA).

### Determining *Cryptosporidium*-specific fecal and serum antibody responses by ELISA.

*Cryptosporidium*-specific antibodies were measured in fecal material and sera from infected mice using enzyme linked immunosorbent assay (ELISAs). Fresh feces and blood samples were collected from each mouse before immunizations and before and after challenge to test for a *Cryptosporidium*-specific IgA or IgG response. Fresh fecal pellets collected from each mouse were suspended in 5 volumes of PBS with protease inhibitor (Sigma catalog number P2714), homogenized, and centrifuged at 18,000 × *g* for 3 min. Supernatant was collected and stored at −20°C. Serum was separated from whole blood by centrifugation and stored at −20°C. *Cryptosporidium*-specific IgA and IgG were detected in the supernatant from fecal sample homogenates and in serum, respectively, using a previously described ELISA protocol (13). *Cryptosporidium* antigen was prepared as previously described (35). Briefly, bleached and washed oocysts were excysted in 1.5% taurocholic acid in sterile PBS for 1 h at 37°C. Supernatant containing shed antigen was collected after centrifugation. The pelleted fraction was additionally processed by repeated freeze and thaw cycles and sonication (Qsonica CL5, Qsonica Sonicators, USA) with 30 cycles of 20 s each. After sonication, supernatant and pelleted fractions were combined. Antigens were stored at −20°C. *Cryptosporidium*-specific IgA and IgG bound to C. parvum antigen were detected with a horseradish peroxidase anti-mouse IgA (ThermoFisher catalog number 62) and anti-mouse IgG (Southern Biotechnology catalog number 1030-05), respectively.

### Statistical analysis.

All data were analyzed using GraphPad Prism version 9.0.1. The Mann-Whitney *t* test, Kruskal-Wallis ANOVA, and a one-way ANOVA with Dunn’s or Dunnett's multiple-comparison test were used to compare groups, as indicated in the figure legends. All results are expressed as means ± standard deviations (SD). *P* values of ≤0.05 were considered statistically significant (*, *P* < 0.05; **, *P* < 0.01; ***, *P* < 0.001; ****, *P* < 0.0001).

### Ethical approval.

The experiments were conducted in accordance with the National Research Council's Guide for the Care and Use of Laboratory Animals (42) and under the Tufts University Institutional Animal Care and Use Committee, protocols G2017-107 and G2020-74.

### Data availability.

All relevant data are provided here.
