# Supplementary material for: Development of Two Mouse Models for Vaccine Evaluation against Cryptosporidiosis
Source: Infect Immun. 2022 Jun 23;90(7):e00127-22. doi: 10.1128/iai.00127-22 (PMC9302090; doi:10.1128/iai.00127-22)
Supplement: Supplemental file 1 — Fig. S1 and S2. Download iai.00127-22-s0001.pdf, PDF file, 0.2 MB [file iai.00127-22-s0001.pdf]

Supplementary Information

Development of Two Mouse Models for Vaccine Evaluation Against Cryptosporidiosis

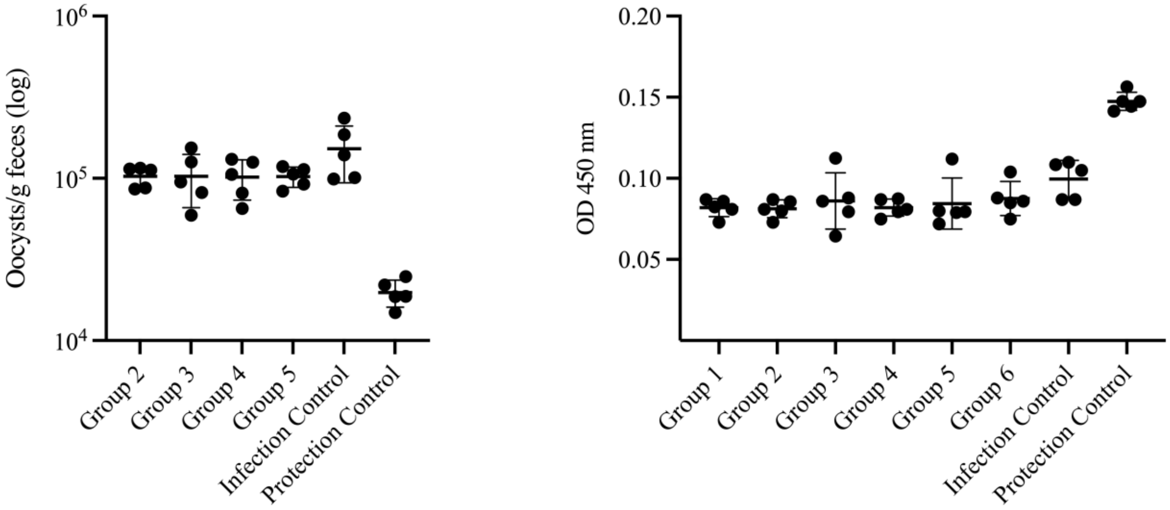

Supplementary Figure S1. Effect of mRNA immunization on oocyst excretion of *C. tyzzeri* (*Ct*) infection. C57BL/6 mice were immunized 3 times with 2-3 weeks intervals with mRNAs and *Ct* oocysts as indicated in Table 1. Two weeks after the third immunization, mice were challenged orally with 10^5 *Ct* oocysts (A) shows the mean oocyst shedding by Group 2, 3, 4, 5 immunized with mRNA combinations, infection and protection control mice. The values are log oocyst concentrations expressed as number of oocysts per gram of feces as quantified by flow cytometry. Mice were individually sampled on 7 timepoints 3-16 days post primary infection and post challenge. The oocyst excreted by Groups 2-5 mice were indistinguishable from the infection control mice. (B) Fecal *Cryptosporidium*-specific IgG in mice. Samples were collected on day 0 post challenge with *Ct* oocysts. Values indicate optical density (OD) measured at 450

nm absorbance. Each data point is mean OD based on duplicates at 1:100 serum dilution. For both graphs, values represent the means for five mice. Error bars show SD.

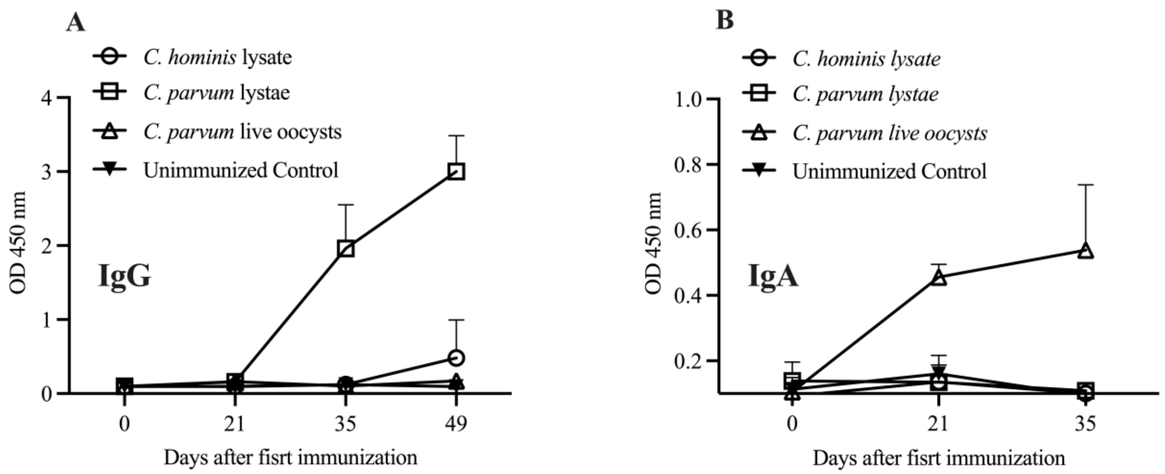

Supplementary Figure S2. The outcome of three IP immunizations with *C. hominis* (*Ch*) or *C. parvum* (*Cp*) lysates in BALB/c mice. Graphs show IgG (A) and IgA (B) in serum (at 1:500 dilution) and fecal samples, respectively. Samples were collected on day 0-before first immunization, 21-before second immunization, 35-before third immunization and 49-before oral challenge with *Ct* oocysts. (A) Mice immunized with *Cp* lysate had elevated *Cryptosporidium*-specific IgG after the second immunization, while unimmunized and mice immunized with *Ch* lysate and *Cp* live oocysts did not generate detectable serum IgG. (B) Immunizations with *Ch* or *Cp* lysates did not result in production of fecal IgA. Values indicate optical density (OD) measured at 450 nm absorbance. Mice were sampled individually, n=5 per immunized group and n=4 for unimmunized mice. Error bars show SD.
